# The Link Between Ferroptosis and Cardiovascular Diseases: A Novel Target for Treatment

**DOI:** 10.3389/fcvm.2021.710963

**Published:** 2021-07-22

**Authors:** Huilin Hu, Yunqing Chen, Lele Jing, Changlin Zhai, Liang Shen

**Affiliations:** ^1^Department of Cardiology, The Affiliated Hospital of Jiaxing University, Zhejiang, China; ^2^Department of Infection, The Affiliated Hospital of Jiaxing University, Zhejiang, China

**Keywords:** cardiovascular diseases, ferroptosis, iron, therapeutic target, lipid peroxidation

## Abstract

Ferroptosis is an iron-dependent cell death, which is characterized by iron overload and lipid peroxidation. Ferroptosis is distinct from apoptosis, necroptosis, autophagy, and other types of cell death in morphology and function. Ferroptosis is regulated by a variety of factors and controlled by several mechanisms, including mitochondrial activity and metabolism of iron, lipid, and amino acids. Accumulating evidence shows that ferroptosis is closely related to a majority of cardiovascular diseases (CVDs), including cardiomyopathy, myocardial infarction, ischemia/reperfusion injury, heart failure, and atherosclerosis. This review summarizes the current status of ferroptosis and discusses ferroptosis as a potential therapeutic target for CVDs.

## Introduction

Cell death plays a vital role throughout the whole life stage. There are two main types of cell death, accidental cell death (ACD) and regulated cell death (RCD) (1). Apoptosis was the first discovered manner of RCD (2). Since then, a growing number of RCD forms were unveiled, including mitochondrial permeability transition (MPT)-derived necrosis, necroptosis, pyroptosis, and ferroptosis (3–7). Ferroptosis is a new type of RCD proposed in 2012 (6), which is induced by the accumulation of iron-triggered lipid peroxidation. Ferroptosis is apparently distinct from apoptosis, necroptosis, and autophagy in several aspects (Table 1). Over the past few years, the research field of ferroptosis has been extraordinary expanded; various mechanisms and regulators were identified in the process of ferroptosis. Ferroptosis is also implicated in an amount of diseases, such as cancer, neurodegenerative diseases, and ischemia–reperfusion injury (8–12).

**Table 1:** Characteristics of different types of cell death.

**Type of cell death**	**Morphological characteristics**	**Biochemical features**	**Detection**	**Regulation**
Ferroptosis	Small mitochondria, increased mitochondrial membrane densities, breakdown of cristae, and outer membrane rupture	Iron overload and lipid peroxidation	Transmission electron microscope,Phen Green SK probe, C11-BODIPY probe, GPX4, Ptgs2	Positive regulator: RAS, NOX, p53, ACSL4, Hmox1, NCOA4 Negative regulator: GPX4, Nrf2, HSPB1, SLC7A11, FSP1
Apoptosis	cell shrinkage, membrane blebbing, chromatin condensation, and formation of apoptotic bodies	DNA fragmentation	Tunel assay, Cytc, cleaved caspase-3, cleaved caspase-9	Positive regulator: Bax family, p53 Negative regulator: Bcl-2 family
Necroptosis	Cytoplasm and organelles swelling, plasma membrane rupture	ROS production, damage-associated molecular patterns (DAMPs) release	RIPK1, RIPK3 and MLKL phosphorylation	Positive regulator: RIPK1, RIPK3, MLKL Negative regulator: Flotillin, syntenin-1
Autophagy	accumulation of double-membrane vesicles	Increased lysosomal activity	Transmission electron microscope, LC3-I, LC3-II	Positive regulator: ATG family, Beclin 1 Negative regulator: mTOR

Recently, ferroptosis has been discovered to contribute to the occurrence and progression of a variety of cardiovascular diseases (CVDs), such as cardiomyopathy, myocardial infarction (MI), ischemia/reperfusion injury (IRI), heart failure (HF), and atherosclerosis (AS). In this review, we summarize the regulatory mechanisms of ferroptosis and discuss the impact of ferroptosis on CVDs.

## Overview of Ferroptosis

Ferroptosis is an distinctive type of non-apoptotic cell death defined by Dixon in 2012 (6), it is an iron-dependent form of RCD, which is characterized by imbalance of iron homeostasis and accumulation of lipid reactive oxygen species (ROS). Actually, ferroptotic cell death can be traced back to the 1950s. Eagle found that deficiency of cystine inhibits cell growth and causes cell death with a distinct morphology; endogenous synthesis of cysteine prevents cells from such cell death (13–15). Following studies further confirmed the importance of cystine deprivation in the process of cell death (16, 17). In 2003, Dolma et al. (18) discovered that a new compound named erastin applied a selectively lethal effect on human foreskin fibroblasts (BJeLR cells) with the expression of the engineered mutant RAS oncogene, but the type of cell death was different from what had been noticed before, with no nuclear morphological changes and DNA breakup. Subsequently, studies further demonstrated that the cell death manner mentioned above could be suppressed by iron chelators and initiated by Ras-selective lethal small molecule (RSL) (19). Then, in 2012, Dixon et al. finally proposed that the non-apoptotic form of cell death was named ferroptosis.

**Figure 1:**
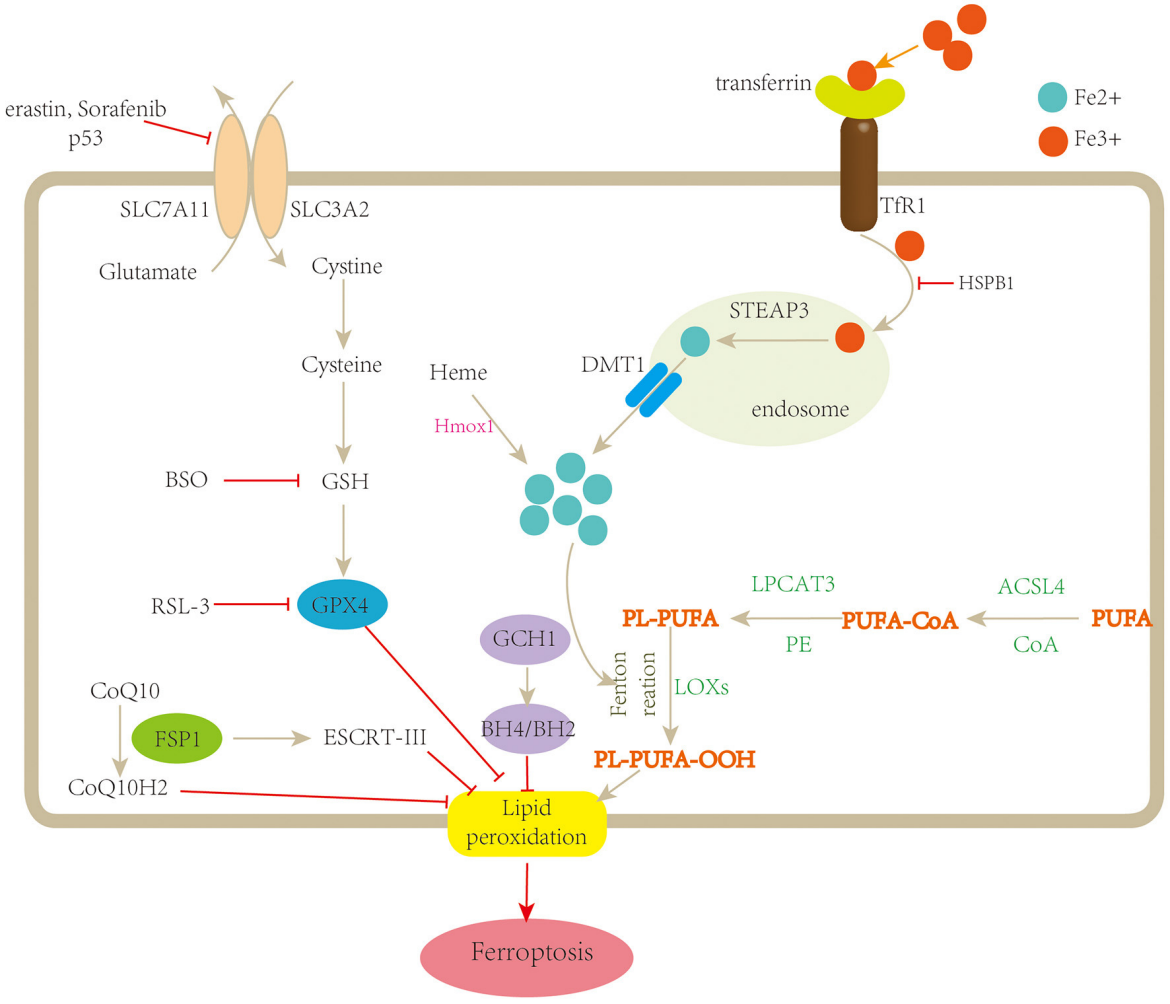
Schematic description of the mechanisms of ferroptosis. Ferroptosis is a type of regulated cell death characterized by iron overload and lipid peroxidation. Fe^3+^ is transferred into the cell by TfR1, then converted to Fe^2+^ in the endosome, and released from the endosome by divalent metal transporter 1 (DMT1). Fenton reaction promotes lipid peroxidation by activating lipoxygenases (LOXs). Cystine is uptaken by system xc^−^ for the synthesis of GSH, which further enhances the anti-lipid peroxidation activity of GPX4. In addition, FSP1-CoQ10 and GCH1-BH4/BH2 are two parallel GPX4-independent pathways in the suppression of ferroptosis. TfR1, transferrin receptor 1; STEAP3, STEAP family member 3; Hmox1, heme oxygenase-1; BSO, buthionine sulfoximine; PUFA, polyunsaturated fatty acid; CoA, coenzyme A; PL, lysophosphatide; ACSL4, acyl-CoA synthetase long-chain family member 4; LPCAT3, lysophosphatidylcholine acyltransferase 3; LOOH, polyunsaturated fatty acid hydroperoxides; GSH, glutathione; SLC7A11, solute carrier family 7 member 11; SLC3A2, solute carrier family 3 member 2; FSP1, ferroptosis suppressor protein1; ESCRT-III, endosomal sorting complex required for transport-III; GCH-1, guanosine triphosphate cyclohydrolase 1; BH4/BH2, tetrahydrobiopterin/dihydrobiopterin.

Unlike cell shrinkage, chromatin condensation and apoptotic body formation presented in apoptosis, cytoplasm, and organelle swelling and cell membrane rupture seen in necrosis, classical double-membrane enclosed vesicles observed in autophagy, pyroptosome assembly, and membrane pore formation detected in pyroptosis and ferroptosis are morphologically, biochemically, and genetically different from these types of RCD. The morphological characteristics of ferroptosis include the shrunken mitochondria with increased mitochondrial membrane density as well as degeneration and breakdown of mitochondrial crista, while the morphology of the nucleus remains unchanged. Ferroptosis can be induced by many chemical compounds and drugs and regulated by a variety of proteins and genes (Table 2). The ferroptosis process also involve multiple metabolic pathways, including iron metabolism, and metabolism of amino acids and lipids (Figure 1).

**Table 2:** Factors involved in ferroptosis regulation.

**Regulator**	**Effects**	**References**
**Positive regulator**		
NCOA4	Mediates iron metabolism	(20, 21)
Hmox1	Mediates heme catabolism	(22)
LPCAT3	Mediateds phospholipid remodeling	(23, 24)
ACSL4	Mediates phospholipid metabolism	(23)
15-LOX	Catalyze the dioxygenation of PUFAs	(25, 26)
p53	Inhibits SLC7A11 expression	(27, 28)
**Negative regulator**		
CISD1 and 2	Regulate mitochondrial iron	(29, 30)
HSPB1	Regulates iron uptake	(31)
GPX4	Reduces phospholipid hydroperoxide	(32)
FSP1	Reduces phospholipid hydroperoxide	(33, 34)
GCH1	Mediates production of nitric oxide and aromatic amino acids	(35)
Nrf2	Maintains oxidative homeostasis	(36)
SLC7A11	Mediates cystine uptake and glutamate release	(37)

*NCOA4, Nuclear receptor coactivator 4; Hmox1, Heme oxygenase 1; LPCTA3, lysophosphatidylcholine acyltransferase 3; ACSL4, Acyl-CoA synthetase long- chain family member 4; 15-LOX, 15-lipoxygenases; CISD, CDGSH iron sufur domain; HSPB1, heat shock protein beta-1; GPX4, glutathione peroxidase 4; FSP1, ferroptosis suppressor protein1; GCH1, guanosine triphosphate cyclohydrolase 1; Nrf2, nuclear factor erythroid 2- related factor 2; SLC7A11, cystine/glutamate antiporter solute carrier family 7 member 11*.

## Mechanism of Ferroptosis

### Iron Metabolism and Ferroptosis

Iron homeostasis is necessary during various biological processes and essential for cell viability. Both iron overload and deficiency cause pathological conditions. Ferroptosis is a type of RCD mediated by excessive iron; iron excess causes lipid peroxidation and triggers cell death.

Although the exact mechanisms between iron metabolism and ferroptosis remain obscure, there is no doubt that iron metabolism plays a key role during ferroptosis. Erastin-induced death is dependent on intracellular iron, but not on other metal ions; co-treatment with the iron chelator deferoxamine suppresses ROS accumulation and ferroptosis (6).

Fe^3+^ is transferred into the cell by transferrin receptor 1 (TfR1), then converted to Fe^2+^ in the endosome by six-transmembrane epithelial antigens of the prostate 3 (STEAP3) and released from the endosome by divalent metal transporter 1 (DMT1). Fe^2+^ is stored in the unstable iron pool (LIP) and ferritin and exported by ferroportin-1 (FPN1) (38). Once the balance between iron absorption, utilization, and recycling was disrupted, free iron ions might accumulate and catalyze the Fenton reaction in the presence of hydrogen peroxide (H_2_O_2_), leading to the formation of lipid peroxides and eventually ferroptosis (39). For instance, silencing the gene encoding TfR 1 can prevent ferroptosis induced by erastin, while depletion of FPN1 increases the sensitivity of cells to ferroptosis (40).

Recent studies have found multiple regulators of iron metabolism involved in the process of ferroptosis. CDGSH iron–sulfur domains 1 and 2 (CISD1 and 2) are both mitochondrial iron regulators; inhibition of CISD1 increases iron-mediated mitochondrial lipid peroxidation, promoting erastin-induced ferroptosis (29), deficiency of CISD2 increases levels of mitochondrial ferrous iron and lipid ROS, facilitating sulfasalazine-induced ferroptosis as well (30). Iron is a cofactor for enzymes involved in many metabolic processes; excess iron could prompt the overproduction of mitochondrial ROS and ferroptosis. Ferritinophagy is a critical mechanism to maintain iron homeostasis in cells. It could induce ferroptosis by promoting labile iron overload and lipid peroxidation (41). Impaired ferritinophagy is related to the resistance of ferroptosis in senescent cells (42); during renal IRI, cold-inducible RNA-binding protein (CIRIB) promotes ferroptosis via activating ferritinophagy (43). Moreover, in cancer cells, ferritinophagy is required for the initiation of ferroptosis through bromodomain protein BRD4 inhibitor (44), and it is also involved in sepsis-induced cardiac injury and zinc oxide nanoparticle (ZnONP)-induced vascular endothelial cytotoxicity (45, 46). Nuclear receptor coactivator 4 (NCOA4) is a cargo receptor for the degradation of ferritin. It maintains intracellular iron homeostasis by mediating ferritinophagy as well (20, 21). Silencing of NCOA4 abolishes the accumulation of reactive iron and ROS, and eventual ferroptotic cell death as well, while overexpression of NCOA4 promotes ferroptosis and increases ferritin degradation (47, 48). Heat shock protein beta-1 (HSPB1), a member of small heat shock proteins (HSPs), has the ability to inhibit iron uptake (31). Upregulation of HSPB1 suppresses erastin-induced ferroptosis. HSPB1 phosphorylation protects cells against ferroptosis by reducing iron-dependent production of lipid ROS (49). Hmox1 is an essential enzyme in heme catabolism. Hmox1 knockout mice present iron accumulation in the liver and kidneys, indicating that Hmox1 is involved in iron metabolism. The impact of Hmox1 on ferroptosis is controversial. Inhibition of Hmox1 by ZnPP represses doxorubicin-induced ferroptosis in the myocardium (22). However, in renal proximal tubule cells, knockout of Hmox1 enhances erastin- or RSL3-induced ferroptosis (50), suggesting that the role of Hmox1 in ferroptosis might be context dependent. Ferritin, a spherical heteropolymer formed by 24 subunits of heavy (FTH) and light (FTL) chain, plays a crucial role in iron metabolism by storing extracellular iron (51). In Hippo mutant cells, increased expression of ferritin heavy chain protects against ROS accumulation and inhibits ferroptosis, and loss of cardiac ferritin heavy chain facilitates cardiomyocyte death and heart failure via ferroptosis (52). Most recently, ceruloplasmin (CP), lactotranferrin (LTF), and prominin-2 (PROM2), which mediates oxidation of Fe^2+^ to Fe^3+^, iron transportation and ferritin exportation, respectively, are all demonstrated to be involved in the process of ferroptosis (53–55). All these studies suggest that iron metabolism plays a crucial role during ferroptosis.

### Lipid Peroxidation and Ferroptosis

Lipid peroxide accumulation is the main feature of ferroptosis. Accumulation of lipid peroxides degrades into reactive toxic aldehydes or hydroxy fatty acids, such as malondialdehyde (MDA), which induces cell toxicity and initiates cell death cascades. Polyunsaturated fatty acids (PUFAs) are components of the cell membrane. ROS react with PUFAs in lipid membranes and induce lipid peroxidation. PUFAs are the most susceptible lipids to peroxidation during ferroptosis; lipoxygenases and phosphorylase kinase G2 are two key regulators of lipid peroxidation during ferroptosis (56). In contrast to PUFAs, monounsaturated fatty acids (MUFAs) exert anti-ferroptotic effects by inhibiting lipid peroxidation. In ovarian cancer cells, overexpression of stearoyl-CoA desaturase 1 (SCD1), an essential enzyme in biosynthesis of MUFAs, shows resistance to ferroptosis (57); besides, SCD1 expressed by cancer cells leads to fatty acid (FA) desaturation and contributes to the protection of oxidative stress-induced ferroptosis, eventually promoting tumor recurrence (58).

Acyl-CoA synthetase long-chain family member 4 (ACSL4) is an enzyme involved in phospholipid metabolism, which plays a key role in the synthesis of long-chain PUFA-CoA. Compared with wild-type cells, ACSL4-deficient cells are accompanied by a significant decrease in AA- and AdA-containing phosphatidylethanolamine (PE), while supplementation with exogenous AA/AdA protects the sensitivity to ferroptosis in ACSL4 knockout cells, indicating that ACSL4 is required for the generation of ferroptosis by accumulating oxidized membrane phospholipids (23). In addition to PE, phosphatidylserine (PS) and phosphatidylinositol (PI) are also involved in ferroptosois (59). Lysophosphatidylcholine acyltransferase 3 (LPCAT3) is closed related to phospholipid remodeling. Downregulation of LPCTA3 also contributes to suppression of ferroptosis, but the function of LPCTA3 in ferroptosis might be less efficient than ACSL4 (23, 24). Lipoxygenases (LOXs) are a family of iron-containing enzymes that catalyze the dioxygenation of PUFAs. However, the role of LOXs in lipid peroxidation and ferroptosis is controversial. The specific inhibitor of 12/15-LOX, but not the inhibitors of 5-LOX or COX, protects GPX4-deficient cells against ferroptosis, suggesting that 12/15-LOX is responsible for ferroptosis (25). Moreover, overexpression of 15-LOX promotes lipid peroxidation, while 15-LOX suppressors, but not COX or CYP450 inhibitors, prevent ferroptosis (26). However, in tert-butyl hydroperoxide (TBH)-induced ferroptosis, p53 upregulates 15-LOX, but not 5-LOX or 12-LOX (60), while in another study, depletion of 12-LOX blocks TBH-induced ferroptosis (27). Therefore, the role of LOXs in ferroptosis needs to be further investigated. In addition to LOXs, other oxygenases, such as NOXs and cytochrome P450 oxidoreductase (POR), are also suggested to be involved in ferroptosis (61–63). Additionally, the HMG-CoA reductase pathway might also participate in the process of ferroptosis. Inhibition of HMG-CoA reductase could induce ferroptosis by inactivating GPX4 (64). Most recently, Chen et al. found that the calcium-independent phospholipase iPLA2β suppresses p53-induced ferroptosis via detoxification of peroxidized lipids. Taken together, the modulation of lipid peroxidation might provide novel therapeutic target for ferroptosis-associated disease.

### Mitochondrial Activity and Ferroptosis

Mitochondria play a vital role in the regulation of cellular metabolism and signal transduction, and they are the main organelles of iron, lipid, and amino acid metabolism. The contribution of mitochondria to ferroptosis is controversial. In melanoma cell lines, inhibition of complex I (CI) of the mitochondrial respiratory chain promotes the opening of the mitochondrial permeability transition pore (mPTP) and cellular ROS levels, as well as accumulation of lipid peroxidation and ferroptosis (65). In cysteine-deprivation-induced ferroptosis, cysteine deficiency leads to hyperpolarization of mitochondrial membrane potential and accumulation of lipid ROS. Inhibition of mitochondrial tricarboxylic acid (TCA) cycle or electron transfer chain (ETC) alleviates lipid ROS accumulation and ferroptosis (66). Moreover, in DOX-induced cardiomyopathy, ferroptosis is the major form of RCD and is mainly triggered in the mitochondria (67). Furthermore, Jelinek et al. (68) and Neitemeier et al. (69) both found that knockout or inhibition of BID preserved mitochondrial integrity and function, therefore, protecting cells against ferroptosis, suggesting the mitochondria as a promising therapeutic target for preventing ferroptosis. However, Gaschler et al. found that cell deficiency of the mitochondria remains sensitive to ferroptotic inducers. Additionally, both ferrostatin-1 (Fer-1) and iron chelators are able to prevent erastin-induced ferroptosis in cells depleted of mitochondria, suggesting that the mitochondria are not necessary for ferroptosis (70). More recently, Song et al. demonstrated that mitochondrial protein PDK4 suppressed ferroptosis by blocking pyruvate oxidation and fatty acid production in pancreatic ductal carcinoma cells, indicating the importance of mitochondria-dependent lipid metabolism in ferroptotic cell death (71). Therefore, further studies are needed to determine the relationship between mitochondrial activity and ferroptosis.

### GPX4-Dependent Pathway

System xc^−^/glutathione (GSH)/glutathione peroxidase 4 (GPX4) axis is considered as the main pathway involved in ferroptosis. System xc^−^ is a cystine/glutamate antiporter comprised of two subunits, SLC7A11 and SLC3A2, which enable the exchange of glutamate and cystine across the cell membrane (37). Once uptaken by system xc^−^ and transported into the cell, cystine is reduced to cysteine, which is essential for GSH biosynthesis. GSH is a potent reductant and a cofactor for GPX4. GSH depletion could induce ferroptosis by increasing lipid ROS (72). Inhibiting the activity of system xc^−^ disturbs the combination of GSH and ultimately decreases the activity of GPX4. Moreover, p53 can inhibit system xc^−^ by suppressing the expression of SLC7A11, thus, impacting the activity of GPX4, leading to an accumulation of lipid ROS and ferroptotic cell death (28).

GPX4 is a member of the glutathione peroxidases (GPXs). It has been shown that GPX4 plays an important role in the process of ferroptosis and is regarded as the key regulator (32). Erastin inhibits system xc^−^ and leads to the depletion of GSH, which inhibits the activity of GPX4 and then promotes the formation of ROS and causes ferroptosis, while RSL3 directly inhibits GPX4 without the depletion of GSH (6). Similar to erastin, sorafenib, a multikinase inhibitor approved for the therapy of hepatic carcinoma (73), also induces ferroptosis through the blockage of GSH synthesis (74). In GPX4 knockout mice, Friedmann Angeli et al. demonstrated an important role of the GSH/GPX4 axis in protecting acute renal failure and related ferroptosis (12). Furthermore, they discovered that liproxstatin-1, a potent spiroquinoxalinamine derivative, suppresses ferroptosis in GPX4 deficiency kidney. In lymphocyte T cells, GPX4 depletion also results in membrane lipid peroxide accumulation and ferroptosis (75).

### GPX4-Independent Pathway

Besides the classical GPX4-dependent ferroptotic pathway, recent studies have uncovered a new regulatory pathway independent of GPX4, including the ferroptosis suppressor protein (FSP1)–CoQ_10_ pathway, endosomal sorting complex required for transport-III (ESCRT-III)-mediated membrane repair pathway and guanosine triphosphate cyclohydrolase 1-tetrahydrobiopterin (GCH1-BH4) pathway.

FSP1 was first defined as a p53-responsive gene, once named apoptosis-inducing factor mitochondria-associated 2 (AIFM2). Recently, two studies simultaneously reported that FSP1 is an effective ferroptosis suppressor and protects cells from ferroptotic cell death. Meanwhile, the inhibition of ferroptosis by FSP1 is mediated by CoQ10 (33, 34). ESCRT-III belong to the family of ESCRT complex, which is composed of five subcomplexes and plays a context-dependent role in membrane remodeling. Dai et al. demonstrated that classical ferroptosis activators such as erastin and RSL3 increase the accumulation of ESCRT-III subunits, while knockdown of components of ESCRT-III machinery enhances ferroptosis (76). BH4 is a cofactor involved in the production of aromatic amino acids and nitric oxide. GCH1 is the rate-limiting enzyme for BH4 synthesis. Through using CRISPR-mediated whole-genome activation screens, Kraft et al. found that GCH1 is the most prominent gene involved in the suppression of ferroptosis. Upregulation or silence of GCH1 causes cancer cells correspondingly resistant or sensitive to ferroptosis by controlling endogenous production of the antioxidant BH4, suggesting a distinctive mechanism of ferroptosis protection that is independent of GPX4 (35).

## Ferroptosis and Cardiovascular Diseases

### Ferroptosis and Cardiomyopathy

Cardiomyopathy is a progressive heart disease with multifactorial pathogenesis and high mortality. Cardiomyopathy is always accompanied by cardiomyocyte death, which contributes to pathological ventricular remodeling and heart failure.

Doxorubicin (DOX) is a second-generation anthracycline chemotherapeutic drug that causes cardiotoxicity, leading to cardiomyopathy named DOX-induced cardiomyopathy (77). Fang et al. (22) found that ferroptosis plays a crucial role in the DOX-induced cardiomyopathy. DOX treatment induced a robust increase in prostaglandin endoperoxide synthase 2 (Ptgs2) mRNA in the heart. Mice pretreated with Fer-1 or dexrazoxane (DXZ, the prodrug for iron chelation) significantly improved cardiac function and reduced DOX-induced cardiac injury mortality. RNA sequencing (RNA-seq) analysis showed that Hmox1 was significantly upregulated, accompanied by activation of the Nrf2, and the Nrf2/Hmox1 pathway causes heme degradation and free iron release in the myocardium. Besides, the authors also determined the effects of MitoTEMPO, a mitochondrially targeted antioxidant, and found that MitoTEMPO suppressed lipid peroxidation and cardiac ferroptosis, which eventually attenuate DOX-induced cardiomyopathy.

Tadokoro et al. (67) also investigated the role of ferroptosis in DOX-induced cardiomyopathy and demonstrated that mitochondria-dependent ferroptosis plays a pivotal role in doxorubicin cardiotoxicity. In this study, the authors found that in DOX-induced cardiomyopathy mice and DOX-cultured cardiomyocytes, GPX4 was downregulated, and lipid peroxides were accumulated especially in the mitochondria. Overexpression of GPX4 or iron chelation targeting Fe^2+^ in mitochondria prevented ferroptosis in cultured cardiomyocytes. They concluded that ferroptosis is the major form of RCD in DOX-induced cardiomyopathy, and DOX-induced ferroptosis is triggered in the mitochondria.

In another study, Liu et al. also detected ferroptosis in DOX-induced cardiomyopathy. Suppression of ferroptosis by Fer-1 effectively prevented cardiac injury. Moreover, the authors found that acyl-CoA thioesterase 1 (Acot1), one of the leading-edge core genes, protects the deterioration of DOX-induced cardiomyopathy by suppressing ferroptosis (78).

Diabetic cardiomyopathy (DCM) is described as a specific abnormality of the myocardial structure and function in diabetic patients and increases the risk of HF independent of coronary artery disease and hypertension (79, 80). Cell death is considered as the terminal pathway of cardiomyocytes in the process of DCM (81). As a new RCD form, whether ferroptosis participates in the pathology of DCM is unclear.

ROS are excessively produced in diabetes mellitus and regarded as the predominant pathogenesis for the progression of DCM (82). Since ROS production promotes ferroptosis, it is reasonable to believe that ferroptosis is involved in DCM. Therapeutic inhibition of mitochondrial ROS by MitoTEMPO abrogated high glucose-induced cell death and mitigated myocardial dysfunction in diabetic mice, suggesting that MitoTEMPO might have been a therapeutic strategy for DCM (83).

Nrf2 is a crucial factor in maintaining oxidative homeostasis and has been proposed as a potential therapeutic target for DCM (36). There is a growing number of evidences that indicate that Nrf2 reduces high glucose-induced oxidative damage and prevents the development of DCM. In type 1 diabetic mice, Zang et al. found that Nrf2 knockout inhibits cardiac pathological remodeling and oxidative stress. Meanwhile, chronic type 1 diabetes leads to autophagy deficiency, which promotes Nrf2-mediated ferroptosis, thereby exaggerating the progression of DCM (84). Considering that Nrf2 plays a vital role in regulating genes for glutathione regulation and lipid peroxidation, which are implicated in ferroptosis (85, 86), altered ferroptosis by Nrf2 activators may play an important role in the development of DCM.

Vascular endothelial dysfunction is thought to be one of the major causes of DCM (87). Recently, Luo et al. (88) found that high glucose induces ferroptosis in human umbilical vein endothelial cells (HUVECs) and concluded that ferroptosis is involved in endothelial dysfunction via p53-xCT (the substrate-specific subunit of system xc^−^)–GSH axis, suggesting that ferroptosis might participate in the process of DCM.

### Ferroptosis and Myocardial Infarction

MI defines cardiomyocyte necrosis in a clinical situation consistent with acute myocardial ischemia, which causes ventricular remodeling and HF (89, 90). Myocardial iron is a risk factor for adverse left ventricular (LV) remodeling after MI.

Baba et al. (91) examined the role of cardiac mTOR in iron-mediated cell death and found that mTOR overexpression inhibits ROS production and ferroptosis, while mTOR deficiency promotes iron-induced ferroptosis. Cardiac mTOR might be an effective therapeutic target in acute MI through specifically managing iron homeostasis. However, in diabetic rats, Wang et al. demonstrated that diabetes aggravates myocardial IRI through programmed cell death such as apoptosis and ferroptosis, while AMPK attenuates post-ischemic myocardial injury and ferroptosis (92). Besides, AMPK activation is also involved in energy stress-induced ferroptosis (93). As we know, AMPK might involve mTOR inhibition; hence, the role of mTOR in cardiac ferroptosis needs further investigation.

In addition to mTOR, exosome-mediated ferroptosis was also investigated in myocardial injury. Song et al. (94) demonstrated that ferroptosis was generated in the infarcted myocardium and in cardiomyocyte following hypoxia-induced injury. Human umbilical cord blood-derived MSC (HUCB-MSCs) exosomes inhibit ferroptosis and attenuate myocardial injury by suppressing DMT1 expression. DMT1 is responsible for iron import. Upregulation of DMT1 might increase iron uptake, resulting in lipid peroxidation and ferroptosis (95). In Parkinson's disease mice model, the expression of DMT1 was significantly increased, leading to iron deposition and ferroptosis (96). Besides, overexpression of NF-κB-regulated 1b isoform (1B)/DMT1 contributes to cell death during brain ischemia (97), suggesting that DMT1-mediated ferroptosis might be a therapeutic target for myocardial ischemia.

Oxidative stress is known to contribute the progression of MI. During MI, both glutathione metabolic pathway and ROS pathway were significantly inhibited (98). GPX4 expression was repressed in the early and middle stages of MI, accompanied by Nrf2, and epithelial–mesenchymal transition (EMT) pathways were significantly altered. In particular, the Nrf2–Hmox1 pathway was enhanced in the early and middle stages of MI, and EMT- Zeb1/2 was increased in the early stage of MI. However, not all studies come to the same conclusion. In a study conducted by Tang et al. (99), ferroptosis was found mainly occurring in the phase of myocardial reperfusion. Therefore, further research is needed to explore the association between ferroptosis and MI, and provides a basis for precise therapy of MI.

### Ferroptosis and Myocardial Ischemia/Reperfusion Injury

Myocardial IRI is a pathological event characterized by an initial restriction of blood supply to the myocardium, usually as a result of severe stenosis or thrombosis, followed by restoration of blood flow and concomitant reoxygenation to the ischemic region. The mechanisms of IRI include oxidative stress, calcium overload, mitochondrial dysfunction, inflammation, microvascular dysfunction, and activation of cell death pathways (100). Cell death is frequently associated with IRI; however, IRI-induced cardiac myocyte death has not been fully explicated. Recent studies indicate that ferroptosis might participate in IRI. Compared with sham-operated mice, mice suffered to myocardial I/R had significantly higher levels of cardiac Ptgs2 mRNA expression (22). Pretreatment with Fer-1 and DXZ reduced myocardial infarct size. Besides, liproxstatin-1 decreased mitochondrial ROS production and increased GPX4 protein level in the I/R model (101). Overexpression of USP22 could inhibit ferroptotic cardiomyocyte death to protect against IRI (102). Most recently, Stamenkovic et al. demonstrated that oxidized phosphatidylcholines (OxPCs) increased in the process of myocardial IRI, leading to decreased GPX4 activity and increased ferroptosis. Notably, cell death induced by OxPCs could be suppressed by Fer-1 (103). In another study, the expression of embryonic lethal-abnormal vision-like protein 1 (ELAVL1) was upregulated during myocardial I/R. Knockdown of ELAVL1 decreased ferroptosis and ameliorated IRI. The authors also showed that ELAVL1 was transcriptionally activated by forkhead box C1 (FOXC1), and the process of ferroptosis was modulated by autophagy (104).

Ferroptosis is involved in diabetes myocardial IRI as well. Li et al. (105) investigated the effect of ferroptosis in the process of diabetes myocardial IRI and revealed that inhibition of ferroptosis could reduce endoplasmic reticulum stress and myocardial injury in the rat I/R model and reduce cell injury in H9c2 cells. Additionally, as mentioned above, diabetes exaggerates myocardial IRI through activating Nox2-related oxidative stress and ferroptosis, while suppression of Nox2 protects diabetic rats from myocardial IRI (92), providing a therapeutic regent for myocardial IRI in diabetic patients.

The association between ferroptosis and heart transplantation-related IRI has been investigated as well (106). The results showed that ferroptosis recruited neutrophil to the injured myocardium by releasing damage-associated molecular patterns (DAMPs). Fer-1 reduces cardiomyocyte death and blocks neutrophil recruitment following heart transplantation. Targeting ferroptosis might provide therapeutic strategies for patients who are vulnerable to IRI following restoration of coronary blood flow after heart transplantation.

### Ferroptosis and Heart Failure

HF is a clinical syndrome caused by a structure and/or function cardiac abnormality, leading to a reduced cardiac output and/or elevated intracardiac pressures at rest or during stress (107). Iron homeostasis is important for maintaining the function of cardiomyocytes. Both iron deficiency and overload are associated with HF. Fang et al. (52) discovered that mice lacking ferritin H (Fth) have increased ROS production and developed HF at 6 months of age. High-iron diet caused ferroptosis and myocardial hypertrophy in cardiac Fth loss mice, which was suppressed by Fer-1. They also found that overexpressing Slc7a11 in cardiomyocytes suppressed high-iron diet-induced cardiac ferroptosis and protected against HF.

In aortic banding-induced heart failure, ferroptosis was also observed. Puerarin, an antioxidant reagent, exhibited cardioprotective function by significantly blocking iron overload and lipid peroxidation, indicating that puerarin can alleviate HF partly through ferroptosis mitigation (108). In addition, Chen et al. (109) also investigated the role of ferroptosis in pressure overload-induced HF. The results showed that ferroptosis was detected in HF rats caused by aortic banding. Either TLR4 and NADPH oxidase 4 (NOX4) knock-down through lentiviral delivery of siRNA remarkably inhibited the activation of ferroptosis and relieved the symptoms of HF, suggesting TLR4-NOX4 as a potential therapeutic target for HF by inhibiting ferroptosis-mediated cell death. Most recently, the relationship between circRNA, iron metabolism, and heart failure was also investigated, and the results showed that ferroptosis was increased in transverse aortic constriction (TAC)-induced HF mice. Furthermore, the interrelationships of miR-224-5p with circSnx12 and Fth1 was established. During HF, the expression of Fth1 was downregulated, which, in turn, releases a large amount of ferrous ions and eventually contributes to ROS accumulation and ferroptotic cell death (110).

### Ferroptosis and Atherosclerosis

Atherosclerosis (AS) is a chronic inflammatory disorder characterized by disorders of lipid metabolism. It is well-known that lipid peroxidation plays a significant role in AS. Guo et al. (111) found that overexpressing GPX4 decreased lipid peroxidation and alleviated atherosclerotic lesions in the aorta of ApoE-deficient mice. As excessive lipid peroxidation is the main feature of ferroptosis, it is reasonable to believe that ferroptosis might participate in the initiation and progression of AS. Recently, a study by Bai et al. (112) explored the potential effects of ferroptosis on AS. The results showed that inhibition of ferroptosis could inhibit lipid peroxidation and alleviate high fat food-induced AS lesion in ApoE–/– mice. Additionally, ox-LDL causes mitochondrial damage and downregulates the expressions of SLC7A11 and GPX4 in mouse aortic endothelial cells (MAECs), while Fer-1 could repress ox-LDL-induced lipid peroxidation and endothelial dysfunction in MAECs. They concluded that ferroptosis might be involved in the pathological process of AS and potentially to be a therapeutic target for AS. In addition, ferroptosis is detected in human coronary artery specimens. In the advanced stages of AS, the expression of Ptgs2 and ACSL4 were upregulated, while GPX4 was downregulated. The severity of AS was associated with Ptgs2 and ACSL4 positively and GPX4 negatively. They concluded that ferroptosis and pyroptosis may regulate the occurrence and progress of AS, providing potential target for the prevention and therapy of coronary artery atherosclerosis (113).

### Ferroptosis and Other Cardiovascular Disorders

In sepsis-induced cardiac injury, lipopolysaccharide (LPS) increased the levels of Ptgs2 and lipid ROS, which were attenuated by Fer-1 and DXZ (45). LPS upregulated the expression of NCOA4 and the level of intracellular Fe^2+^; cytoplasmic Fe^2+^ further increased the level of siderofexin (SFXN1) on mitochondrial membrane, which in turn carried cytoplasmic Fe^2+^ into the mitochondria, leading to the generation of mitochondrial ROS and ferroptosis. Ferroptosis might be a therapeutic target for preventing sepsis in the future.

Arrhythmias are particularly common in patients with heart disease. Studies have shown that excessive ROS can cause arrhythmia (114). To the best of our knowledge, ferroptosis has not yet been investigated in arrhythmia. A recent study revealed that iron overload resulted in mitochondrial ROS generation and mitochondrial membrane potential (ΔΨ_m_) depolarization, thus, opening the mPTP, thereby promoting cardiac arrhythmias (115). As we know, mitochondrial ROS generation and ΔΨ_m_ depolarization are features of ferroptosis as well. Targeting ferroptosis might provide a new idea for the treatment of arrhythmia.

Coronavirus disease 2019 (COVID-19), caused by the SARS-CoV-2 virus, has caused a major health problem around the world. Multiple organs affected in COVID-19 include the lungs, kidneys, liver, and heart. As a novel RCD, ferroptosis might be an important cause of cardiac injury in COVID-19 and serves as a new therapeutic target. In one COVID-19 patient, Jacobs et al. discovered lipid peroxidation in the myocardium and concluded that ferroptosis is a detrimental factor in COVID-19 cardiac damage (116). Iron overload, which is associated with inflammation, hypercoagulation, and immune dysfunction, has been considered as a vital player in the pathogenesis of COVID-19, leading to acute respiratory distress syndrome (ARDS), thromboembolic complications, and cardiovascular damage (117, 118). Iron chelators or ferroptosis inhibitors might be potential treatment candidates for COVID-19-related multiple organ failure.

## Conclusion and Perspective

Death of cardiomyocyte results in dramatic impairments to cardiac function and eventually in HF (119); therefore, preventing and reducing cardiomyocyte death is crucial to the improvement of cardiac function. Recently, ferroptosis, an iron-mediated RCD, has received growing attention and is found to be associated with cardiac pathology. Targeting ferroptosis may provide potential treatment strategy for CVDs (Table 3).

**Table 3:** Potential therapeutic strategy for CVDs.

**Reagents**	**Function**	**Diseases**	**References**
Fer-1	Blocks lipid peroxidation	DOX-induced cardiomyopathy, Myocardial IRI, AS, Sepsis-induced cardiac injury	(22, 45, 78, 103, 112)
DXZ	Prevents lipid peroxidation	DOX-induced cardiomyopathy, Myocardial IRI, Sepsis-induced cardiac injury	(22, 45)
Mito TEMPO	Suppress lipid peroxidation	DOX-induced cardiomyopathy, DCM	(22, 83)
Lip-1	Increases GPX4 and reduce ROS	Myocardial IRI	(101)
mTOR	Prevents iron overload	Myocardial infarction	(91)
Puerarin	Blocks iron overload and lipid peroxidation	Heart failure induced by pressure overload	(108)

*Fer-1, ferrostatin-1; DOX, doxorubicin; IRI, ischemia-reperfusion injury; AS, atherosclerosis; DXZ, dexrazoxane; DCM, diabetic cardiomyopathy; Lip-1, liproxstatin-1; GPX4, glutathione peroxidase 4; ROS, reactive oxygen species*.

As discussed above, ferroptosis is involved in the process of DOX-induced cardiomyopathy, diabetic cardiomyopathy, MI, myocardial IRI, HF, AS, sepsis-induced cardiac injury, cardiac arrhythmia, and COVID-19-associated cardiac injury, while the relationship between ferroptosis and other cardiovascular disorders, such as dilated cardiomyopathy, valvular heart disease, myocarditis, and aortic disease (including aortic dissection and aortic aneurysm), has not ever been investigated. Further studies are needed to establish the web between ferroptosis and various CVDs.

Although ferroptosis occurs in various diseases, whether it acts as an automatic response to the stimuli that disrupt the metabolic balance or it is the stimuli that directly interrupt the balance is controversial. In other words, we still have no idea whether ferroptosis is achieved “actively” or “passively.” Further studies are needed to explore the exact association between ferroptosis and the different pathophysiological conditions.

Increasing evidence has shown the crosstalk between ferroptosis and other types of cell death, such as autophagy and apoptosis (21, 28). Whether these various modes of cell death can be integrated into a complete regulatory network remains to be explored. Further elucidating the interrelation between ferroptosis and other types of cell death is important to clarify the mechanisms of ferroptosis and developing treatments.

At present, there are no specific biomarkers for ferroptosis. Since ferroptosis occurs in the early stage of MI and cardiomyopathy, discovery of specific markers of ferroptosis may provide great assistance in early diagnosis of CVDs. Moreover, the cardioprotection of ferroptosis suppressors is just limited in animal studies. How basic research results should be applied in a clinical situation also needs further exploration. Nevertheless, inhibition of ferroptosis is thought to be a potential therapeutic option for CVDs (22, 120).

In conclusion, ferroptosis plays a critical role in the pathogenesis of various CVDs, but its mechanism needs to be further explored. Suppression of ferroptosis is expected to become an effective therapeutic strategy for CVDs.

## Author Contributions

LS designed the study and revised the manuscript. LJ generated the figure and tables. CZ edited the manuscript. HH and YC drafted the manuscript. All authors have read and approved the content of the manuscript.

## Conflict of Interest

The authors declare that the research was conducted in the absence of any commercial or financial relationships that could be construed as a potential conflict of interest.
